# Diagnosis and management of congenital type D esophageal atresia

**DOI:** 10.1007/s00383-023-05519-6

**Published:** 2023-10-10

**Authors:** Cuizhu Feng, Long Li, Yanxia Zhang, Yong Zhao, Jinshi Huang

**Affiliations:** 1 Department of Pediatric Surgery, Capital Institution of Pediatrics, Beijing, People’s Republic of China; 2 Research Unit of Minimally Invasive Pediatric Surgery on Diagnosis and Treatment, Chinese Academy of Medical Sciences 2021RU015, Beijing, China; 3 Department of Neonatal Surgery, Beijing Children’s Hospital, Capital Medical University, National Center of Children’s Health, Beijing, People’s Republic of China

**Keywords:** Type D esophageal atresia, Esophagography, Tracheoscopy, Surgical treatment

## Abstract

This study was performed to describe the current clinical practice and outcomes of type D esophageal atresia. We retrospectively analyzed 10 patients who were diagnosed with type D esophageal atresia and underwent esophageal atresia and tracheoesophageal fistula repair in the Capital Institute of Pediatrics and Beijing Children’s Hospital from January 2017 to May 2022. Ten patients include three newborns and seven non-newborns. Seven (70%) cases were misdiagnosed as type C esophageal atresia before the first operation. Three neonatal children underwent thoracoscopic distal tracheoesophageal fistula ligation and esophageal anastomosis: the proximal tracheoesophageal fistula was simultaneously repaired with thoracoscopy in one of these children, and the proximal tracheoesophageal fistula was not detected under thoracoscopy in the other two children. Among the seven non-neonatal children, one underwent repair of the proximal tracheoesophageal fistula through the chest and the other six underwent repair through the neck. Nine patients were cured, and one died of complications of severe congenital heart disease. Type D esophageal atresia lacks specific clinical manifestations. Misdiagnosis as type C esophageal atresia is the main cause of an unplanned reoperation. Patients without severe malformations have a good prognosis.

## Introduction

Esophageal atresia (EA) is a congenital disease in which the middle esophagus is missing, and it is often associated with one or more tracheoesophageal fistulas (TEFs), The reported incidence is from 1 in 2500 births to 1 in 4500 births [1–4]. Gross classification of the anatomic patterns of EA is often used. Type D EA is defined as EA with a double (proximal and distal) fistula. Though rare, this form is found more often than originally thought. The incidence of type D EA is about 1%. Because of its low incidence rate and lack of specific clinical manifestations, type D EA is often misdiagnosed as type C EA, leading to an unplanned reoperation. How to reduce the misdiagnosis rate and improve the operative success rate are important problems for pediatric surgeons. Following approval by the Capital Institute of Pediatrics and Beijing Children’s Hospital Review Board, we reviewed 10 patients who were diagnosed with type D EA and underwent EA/TEF repair in the Capital Institute of Pediatrics and Beijing Children’s Hospital from January 2017 to May 2022. We also herein discuss the diagnostic and treatment strategies for type D EA.

## Materials and methods

### Patients

Ten patients with type D EA were treated at the Capital Institute of Pediatrics and Beijing Children’s Hospital from January 2017 to May 2022. They comprised seven male and three female patients. According to their age at diagnosis, they were divided into a neonatal group (three patients; age at diagnosis, 1–9 days) and a non-neonatal group (seven patients; age at diagnosis, 1 month to 4 years 7 months).

### Clinical signs

The earliest clinical feature in the neonatal group was usually excessive salivation. Typically, the first feeding was followed by regurgitation, choking, and coughing. Another feature was the inability to pass a feeding or suction catheter through the mouth or nose into the stomach. All children in the non-neonatal group were diagnosed with type C EA in the neonatal period and underwent esophageal anastomosis and TEF repair. The main signs were choking and coughing while feeding, repeated pneumonia, and no weight gain.

### Diagnostic and surgical methods

Esophagography and tracheoscopy were the main methods to detect proximal TEF and diagnose type D EA. The patients in the neonatal group underwent thoracoscopic distal and proximal TEF repair and esophageal anastomosis. If the proximal fistula could not be found by thoracoscopy, the proximal TEF was repaired several weeks later through the neck. The patients in the non-neonatal group underwent proximal TEF repair through the neck or chest.

## Results

The clinical characteristics of the patients in the neonatal and non-neonatal groups are shown in Tables 1 and 2, respectively. In all patients, esophagography with water soluble contrast material was performed in the neonatal period, and a proximal TEF was found in only one patient.

**Table 1:** Clinical characteristics of neonatal group

Patient no.	Sex	Age at diagnosis	Clinical signs	Methods to detect proximal fistula	Tracheoscopy findings	Surgical methods	Result
1	F	3 days	Choking and coughing, inability to pass feeding catheter into stomach	Tracheoscopy	The distal fistula was located near the bulge and was large and concave. The proximal fistula was located approximately 3.6 cm from the bulge and was small and slit-shaped. The Meilan test was positive for both fistulas	1. Thoracoscopic EA/distal TEF repair	Cure
						2. Proximal TEF repair via neck 2 weeks later	
2	F	9 days	Choking and coughing, inability to pass feeding catheter into stomach	Esophagography		Thoracoscopic EA/distal TEF repair; transcervical repair of the proximal TEF was planned several weeks later	Died after discontinuing treatment for severe heart disease
3	M	1 day	Excessive salivation, inability to pass feeding catheter into stomach	Operation		Thoracoscopic EA/distal and proximal TEF repair	Cure

*F* female, *M* male, *EA* esophageal atresia, *TEF* tracheoesophageal fistula

**Table 2:** Clinical characteristics of non-neonatal group

Patient no.	Sex	Age at diagnosis	Clinical signs	Methods to detect proximal fistula	Tracheoscopy findings	Surgical methods	Result
1	M	1 month	Choking and coughing; pneumonia	Tracheoscopy	The proximal fistula was 2 cm from the bulge and positive on the Meilan test. The concave region near the bulge was negative on the Meilan test	Proximal TEF repair via cervicotomy	Cure
2	F	2 months	Choking and coughing; pneumonia	Tracheoscopy	The proximal fistula was 1.5 cm from the glottis and was smaller and slit-shaped. The Meilan test was positive. The concave region near the bulge was negative on the Meilan test	Proximal TEF repair via thoracoscope	Cure
3	M	3 months	Choking and coughing; repeated pneumonia	Tracheoscopy, esophagography	The proximal fistula was 3.5 cm from the bulge and was positive on the Meilan test. The concave region near the bulge was negative on the Meilan test	Proximal TEF repair via cervicotomy	Cure
4	M	5 months	Choking and coughing; repeated pneumonia; no weight gain	Tracheoscopy	The proximal fistula was 3.0 cm from the bulge and positive on the Meilan test. The upper margin of the bulge was abnormally concave, and the Meilan test was negative	Proximal TEF repair via cervicotomy	Cure
5	M	6 months	Choking and coughing; repeated pneumonia	Tracheoscopy	The proximal fistula was 3.5 cm from the bulge and positive on the Meilan test. The abnormal concave region was above the bulge, and the Meilan test was negative	Proximal TEF repair via cervicotomy	Cure
6	M	4 years 7 months	Choking and coughing; repeated pneumonia	Tracheoscopy, esophagography	The proximal fistula was 4.5 cm from the bulge and 2.5 cm from the glottis and positive on the Meilan test. The concave region on the lower trachea was negative on the Meilan test	Proximal TEF repair via cervicotomy	Cure
7	M	2 months	Choking and coughing	Tracheoscopy, esophagography	The proximal fistula was 3 cm from the bulge and positive on the Meilan test. The concave region near the bulge was negative on the Meilan test	Proximal TEF repair via cervicotomy	Cure

*F* female, *M* male, *TEF* tracheoesophageal fistula

### Neonatal group

Patient 1 was diagnosed with type D EA by esophagography. Patient 2 was suspected to have type D EA because the upper blind pouch was thin (0.9-cm diameter). Tracheoscopy was further performed to define the type D EA. Patient 3 was misdiagnosed with type C EA; the proximal fistula was found during the operation, and the diagnosis was revised to type D EA. In Patients 1 and 2, the proximal TEF was not detected by thoracoscopy during EA/TEF repair, and these patients were planned to undergo repair through the neck several weeks later. After 2 weeks, Patient 1 underwent proximal TEF repair through the neck and recovered after surgery. Patient 2 died after discontinuing treatment for severe heart disease. Patient 3 underwent simultaneous repair of the proximal TEF with thoracoscopy. This patient also had anal atresia and underwent a colostomy.

### Non-neonatal group

In seven patients, the missing proximal TEF was found by tracheoscopy and/or esophagography, and their diagnosis was revised to type D EA. One patient underwent repair by thoracoscopy, and the other six underwent repair via the neck.

### Overall population

Nine (90%) patients were cured. During the postoperative follow-up of 2 to 3 years, two cases of anastomosis stricture were cured after balloon expansion guided by gastroscopy. One patient developed relapse of the distal TEF and was cured by thoracoscopic surgery. One patient was cured after anoplasty and closure of the colon stoma.

## Discussion

Congenital EA is a serious digestive tract malformation caused by an esophageal development disorder at 3 to 6 weeks of embryonic development. It is often accompanied by one or more TEFs because of incomplete separation between the esophagus and trachea. Type C EA is the most common, accounting for about 85% to 90% of all cases. Type D EA is characterized by both a distal and proximal TEF and accounts for only 1% of all cases of EA. A few case reports of type D EA are present in the literature [5–7].

The main clinical signs of type D EA after birth are spitting, choking, and inability to pass a feeding or suction catheter through the mouth or nose into the stomach. Because these signs are not different from those of type C EA, it is difficult to distinguish between the types by clinical signs alone.

Esophagography is a routine examination technique for EA. Water soluble contrast material such as iohexol should be used to avoid or reduce the damage caused by inhaling the contrast agent. Contrast agent (1–2 mL) should be slowly injected into the gastric tube. The portion of the gastric tube with the lateral hole must be cut off and advanced into the esophagus for about 10 cm. Esophagography is performed with the patient in different positions to understand the length and width of the upper esophageal pouch and the potential presence of a TEF. It has been suggested that the prone position can be used for esophagography and that the fistula can be more easily displayed in this position [8]. After examination, the contrast agent in the upper pouch should be pumped as soon as possible. The position of the distal TEF shows few changes, so the length of the missing esophagus can be predicted according to the position of the upper esophageal pouch. It is difficult to identify the proximal TEF by esophagography. In the present study, all 10 patients underwent esophagography, but the proximal TEF was found in the neonatal period in only one patient. One newborn was considered to possibly have type D EA because the esophagogram showed that the proximal pouch was thin (0.9 cm in diameter). Our clinical experience has shown that the proximal pouch diameter is about 1.3 cm in type C EA. In the above-mentioned patient, type D EA was finally confirmed by bronchoscopy. Therefore, the possibility of having a proximal fistula should be considered when esophagography shows a thin proximal pouch.

Bronchoscopy has obvious advantages over other examination methods for the diagnosis of TEF [9, 10]. Eight (80%) children in the present study were definitively diagnosed by bronchoscopy. At the same time, the distance can be measured between the fistula and the glottis, which can provide a basis for the operative approach through the chest or neck. However, because of the low incidence of type D EA, there are still many disputes regarding whether every child with congenital EA requires bronchoscopy. We suggest the use of bronchoscopy when the esophagogram shows a thin proximal pouch and when the child has recurrent pneumonia and no weight gain after operative treatment of type C EA [11, 12]. Repeated bronchoscopy can be performed when the diagnosis is unclear. To improve the recognition rate of the fistula, we injected methylene blue into the esophagus through the gastric tube and observed whether the trachea was stained blue during tracheoscopy [13].

Whether to repair the proximal fistula in type D EA through the chest or neck has been a topic of debate. Parolini et al. [14] systematically evaluated 90 cases of TEF in 17 articles and concluded that the neck approach could be used for fistulas above the T2 level and that transthoracic surgery could be used for fistulas at the T2 to T4 level. Another study indicated that fistulas at the T2 to T3 level could be repaired through the neck and that fewer complications and a lower recurrence rate occurred after neck surgery [15]. The diagnostic rate by esophagography in the present study was low, and the fistula positioning mainly depended on bronchoscopy to measure the distance from the fistula to the tracheal lung protrusion and from the fistula to the glottis. The correlation between this distance and the thoracic vertebra requires further examination. The present study suggests that the fistula near the tracheal bulge can be repaired by transthoracic surgery and that the fistula closer to the glottis can be repaired through the neck. An operation through the neck is easier than transthoracic surgery. The overall treatment was satisfactory, and no recurrence after proximal fistula repair occurred in this study.

During surgical repair of type D EA, we observed that the proximal TEF was short and thin, and all were located in the proximal esophageal lateral wall but not at the end of the esophagus, as described in traditional textbooks. This finding explains the low positive rate of esophagography and provides an anatomical basis for intraoperative identification of fistulas. The surgical method also varied based on the different morphology of the proximal and distal fistulas. The distal TEF is located at the end of the esophagus, and it is thick and easy to discern. It can be directly cut off after closure by titanium endoclips [16]. The proximal TEF is short and thin. It is recommended to preserve as many tissues as possible on the tracheal side to facilitate repair.

Type D EA lacks specific clinical manifestations. Misdiagnosis as type C EA is the main cause of an unplanned reoperation. Tracheoscopy is an important examination method for confirmation of type D EA. Patients without severe malformations have a good prognosis.
